# Impact of network aided platforms as educational tools on academic performance and attitude of pharmacology students

**DOI:** 10.12669/pjms.336.13290

**Published:** 2017

**Authors:** Aftab Ahmed Khan, Adel Zia Siddiqui, Syed Fareed Mohsin, Mohammed Mahmoud Al Momani, Eraj Humayun Mirza

**Affiliations:** 1 Aftab Ahmed Khan, MSc, M.Bioeth, B.D.S. Researcher, Dental Biomaterials Research Chair, College of Applied Medical Sciences, King Saud University, Riyadh 11433, Saudi Arabia; 2 Adel Zia Siddiqui, MSc, B.D.S. Associate Professor, Dept. of Dental Materials Sciences, Baqai Dental College, Baqai Medical University, 51 Deh Tor, Toll Plaza, Super Highway, Gadap Road, Karachi 74600; Pakistan; 3 Syed Fareed Mohsin, PhD., MSc, MFD RCSI, MFDS RCPSG, B.D.S. Associate Professor, Dept. of Oral Pathology, College of Dentistry, Qassim University, Saudi Arabia; 4 Mohammed Mahmoud Al Momani, PhD., B.Sc. Assistant Professor, Dept. of ommunity Health Sciences, College of Applied Medical Sciences, King Saud University, Riyadh 11433, Saudi Arabia; 5 Eraj Humayun Mirza, Ph.D., M.Sc., B.Sc. Assistant Professor, Dept. of Biomedical Engineering, Sir Syed University of Engineering and Technology, Karachi, Pakistan

**Keywords:** Academic grades, Learning management system, Mobile learning, Social networking

## Abstract

**Objective:**

This cross-sectional study aimed to examine the impact of learning management system and WhatsApp application as educational tools on students’ academic achievement and attitude.

**Methods:**

The sample population was the students of six medical colleges of Riyadh, Saudi Arabia attending Medical Pharmacology’s semester course in Bachelor of Medicine, Bachelor of Surgery (MBBS) program from September 2016 to January 2017. An exploratory approach was adopted based on a comparison between students exposed to only in-class lectures (Group-N), in-class lectures together with WhatsApp platform to disseminate the lecture slides (Group-W) and students group with in-class lectures facility blended with Learning Management System (LMS) and WhatsApp platform (Group-WL). The students’ grades were assessed using unified multiple choice questions at the end of the semester. Data were analyzed using descriptive statistics and Pearson correlation (p<0.01).

**Results:**

Using learning management system (LMS) and/or WhatsApp messenger tool showed a significant positive correlation in improving students’ grades. Additionally, use of WhatsApp enhances students’ in-class attendance though statistically insignificant.

**Conclusion:**

The results are pivotal for a paradigm shift of in-class lectures and discussion to mobile learning (M-learning). M-learning through WhatsApp may be as an alternative, innovative, and collaborative tool in achieving the required goals in medical education.

## INTRODUCTION

Technological innovations such as laptops and smartphones have greatly affected many aspects of our lives.^1^ Smartphones have become an essential gadget these days. Since the popularity of smartphones, many social networking sites and applications were increased to great extent. Social networking is gaining momentum in the realm of communication and information. However, some people consider social networking sites or applications as frivolous time wasting and distraction from work, and their effect on physical and mental health such as delaying or skipping meals, improper rest, depression and lethargic attitude cannot be over ruled.^2^ Concurrently, social networking platforms such as 2go, WhatsApp Messenger, facebook, and twitter are impacting positively in learners’ progress and knowledge.^3^ Social connections between learners are considered pivotal to construct and share knowledge.^4^,^5^ During the recent technological years, the social networking applications in mobile phones or devices have provided people a platform to reach and exchange information. The unique features of these social networking sites or applications are their compatibility to exchange information, enhance communication and relationship.^6^

E-learning through these social networking sites is a new concept covering novel learning processes and methods.^4^ It provides students expanded options and opportunities in the context of online learning.^7^ A variety of applications such as discussion forums^8^, images, audio and video sharing are possible through these social networking sites or applications.^9^ The activities via social interactions between online students construct awareness and knowledge.^10^

Technically, WhatsApp Messenger can be regarded as a social networking application that allows people to access a great deal of information swiftly.^11^ A communication portal that has rapidly changed the way people communicate.^12^ WhatsApp is a Smartphone application that operates on nearly all current types of devices and operating systems with an active internet connection.^11^ The application has been on the market since 2010. The distinctive feature of WhatsApp Messenger is the option to create a group and to communicate within its boundaries.

The present cross-sectional exploratory study aimed to assess whether using WhatsApp has significant effects on improving the grades and attitudes of students who were enrolled in medical pharmacology course under medical doctor degree among various medical colleges in Riyadh. Furthermore, current study explores student performance and attitude if they are taught in a conventional (face-to-face) approach in the classroom or effectiveness of blended learning management system with WhatsApp. As this phenomenon is quite new and has not yet, to our knowledge, been explored comprehensively among medical students, we, therefore, piloted an exploratory research.

## METHODS

The present cross-sectional analytical study divided study samples according to learning platform in three groups, which are as follows: Group-WL; comprised of students of the two medical colleges followed in-class lectures together with experimental research approach by adopting Learning Management System (LMS) in addition to WhatsApp platform to disseminate the lecture slides and to discuss the foregoing lecture. Group-W; comprised of the students from two other colleges which followed in-class face-to-face activities and WhatsApp activities. The last group i.e., the control group or Group-N; comprised of the students of the remaining two colleges that followed entirely in-class lecturing by the instructor and its discussion only within the lecture time in the classroom. The control group contained 25 students. Whereas experimental Group-W and Group-WL had 21 and 26 students, respectively (Total=72, M=32, F=40). The details are presented in Table-I.

**Table-I:** Division of gender among study groups based on different learning platforms.

	*N*	*W*	*WL*
Male	12	8	12
Female	13	13	14

***Key: N:*** in-class lectures group, ***W:*** WhatsApp in addition to in-class lectures group, ***WL:*** WhatsApp together with learning management system in addition to in-class lectures.

Hence, a total of six medical colleges (3 for male, 3 for female) of Riyadh, Saudi Arabia were selected at random. The sample size was the students (aged 17 to 20) of the MBBS program who were attending the “medical pharmacology” semester course conducted during September 2016 to January 2017. Prior approval was acquired from the head of the institutes and was assured of keeping the name of the institute confidential. Student names were not taken neither their personal contact details were taken. Students were coded as serial numbers. The course content for all the six colleges was the same and each college had a unified multiple-choice question exam at the end of the course. Based on 50 multiple choice questions in the semester exam, the grades of the students were assessed and correlated with the learning facilities available to the students. Only those students were included in this study who were enrolled in the pharmacology course of MBBS program from the start and appeared in the final exam. Students who were enrolled in the middle or dropped the course in between or never appeared in the final exam were excluded. Furthermore, the students of other programs, e.g. B.D.S. or Pharm.D who were appearing in the pharmacology course, were excluded from this study.

Parameters recorded for the intervention were, number of students enrolled in each college, male to female student ratio. Marks obtained in the final exam and attendance throughout the semester. Medium of instruction in all the classes was English. All faculty members were permanent teachers of respective institutes.

The study protocol was approved by the institutional review board of College of Applied Medical Sciences, King Saud University (IRB No. 16-55).

### Statistical analysis

All the collected data were compiled in SPSS v21, IBM. Descriptive statistics were performed and the correlation was obtained using Pearson method. Values were presented as mean ± standard deviation. Any significant difference is reported at a p-value of 0.01.

## RESULTS

Pearson correlation analysis is presented in Table-II. A significant positive relationship between method of teaching (Groups: N, W, and WL) and the marks obtained, r(72) = 0.56, p=0.01 can be observed. On the contrary, there exists no relationship between groups versus attendance; neither there is any impact of attendance on marks.

**Table-II:** Pearson correlation among groups vs. attendance vs. marks.

		*Attendance*	*Marks*	*Group*
Attendance	Pearson		0.113	-0.001
	Correlation	1	0.343	0.995
	Sig. (2-tailed) N	72	72	72
Marks	Pearson	0.113		0.560**
	Correlation	0.343	1	.000
	Sig. (2-tailed) N	72	72	72
Group	Pearson	-0.001	0.560**	
	Correlation	0.995	0.000	1
	Sig. (2-tailed) N	72	72	72

** . Correlation is significant at the 0.01 level (2-tailed).

**Fig.1:**
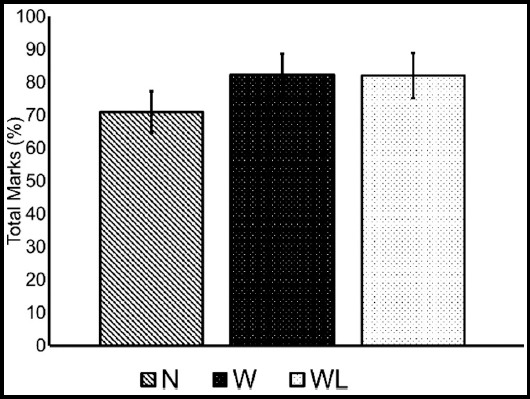
Aggregate of total marks obtained in the final exam via students in all the colleges, grouped according to teaching methodology.

Results in Fig.1 reveal the aggregate percentage achieved by the students of the three groups under study. The mean scores of the three groups show a statistical difference between the Group-N and Group-W. However, no statistical difference was observed in Group-W and Group-WL. On the whole, the students of the Group-WL obtained higher grades followed by Group-W. The Group-N showed the least grades of all the groups.

**Fig.2:**
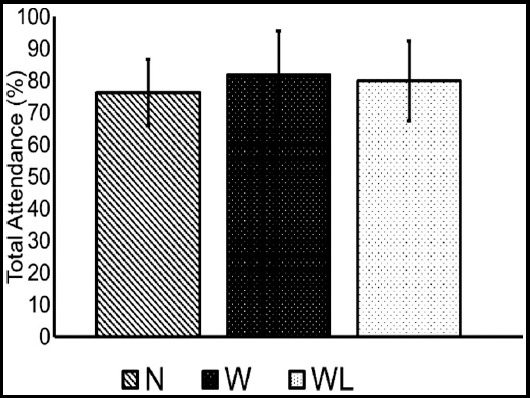
Average of attendance of students in the course in all the colleges, grouped according to teaching methodology.

The bar chart in Fig.2 presents the effect of teaching methodology on the class attendance. The results indicate increased attendance percentage for the Group-W and Group-WL. However, the difference between the studies groups was not statistically significant.

**Fig.3:**
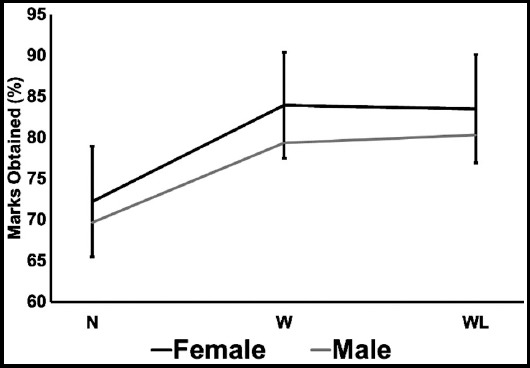
Correlation of Males with Females as per marks obtained (%) with respect to the methodology adopted for studies.

The correlation statistics of marks obtained between males and females is presented in Fig.3. It can be inferred that there was a positive correlation among all the teaching methodologies. Though all the female students obtained higher marks than male counterparts, it is to be noted that none of the groups had a significant difference among them.

## DISCUSSION

The inclination towards E-learning or more precisely Mobile learning (M-learning) is increasing gradually among students and between students and tutor.^13^ The present exploratory study determined the viability and efficiency of novel learning platforms such as LMS and WhatsApp. In addition, the implications of WhatsApp application on overall students’ attendance were looked. Our study set up was medical colleges of Riyadh, Saudi Arabia and thus the socioeconomic status as a barrier in students owning a smartphone is nullified.

The researchers have investigated the studies related to the impact of M-learning on student`s achievements and attitude. However, within medicine, there appears to have been no available data on the effect of M-learning. The strength of this study lies in the objective analysis of the grades which shows that students’ involvement in LMS and WhatsApp group or WhatsApp alone has a significant influence on academic performance. Our results suggest that blended synchronous instruction i.e., in-class lectures with LMS facility and WhatsApp platform or in-class lectures with WhatsApp platform generated a technologically practical approach to solve a problem as a role player. Moreover, it provided the peers a practical setting where complex academic matters can be discussed, simple availability of resources irrespective of distance. Lastly, this platform also created an informal learning system that students may utilize to discover new ideas which eventually leads to intuitive learning process.^14^

Nonetheless, mobile learning cannot substitute the orthodox learning method *i.e*., in-class lectures and discussion. However, it can unquestionably be a supplement for a learner to learn anything, anytime and anywhere.^15^,^16^ Although, the control group (N) still managed to acquire knowledge through the traditional method of teaching it appears that assistance of LMS and WhatsApp could strongly benefit the students in their learning process.

Surprisingly, statistically insignificant differences were observed among W and WL groups. Although the LMS provides a platform where a collaborative and interactive learning activities can be achieved.^17^ However, the availability of online course content without the restrictions and flexibility of online access to information and knowledge might result in a negative influence on learning performance as it solely depends on the students to access the information, whereas the students in W group involuntarily take interest and indulge themselves in activities being conducted in the group. Moreover, the use of LMS as administrative tool in comparison to WhatsApp as collaborative tool might have improved student-teacher interaction resulting in improved learning experiences.^18^-^20^

Another result that warrants mentioning is the statistically insignificant effect of WhatsApp in-class lecture reminder a day before the class. Contrary to expectations, no significant relation was found between N, W and WL groups on students’ in-class attendance. Regardless of expectations, it seems that strategy to prompt students for pre-lecture reminder through WhatsApp effectively increased the overall students’ attendance in class for lectures in the groups W and WL. However, the statistically insignificant difference in students’ attendance with a positive correlation between WhatsApp group and without WhatsApp group may indicate that a larger sample size could more validly reflect the mean population.

Interestingly the marks obtained by female students remained higher specifically in the W and WL groups. This incremental result can be attributed to females being more addicted to mobiles and the internet. Moreover, a similar pattern of addiction was observed by Chiu S.I and co-workers, where they reported that females preferred online communication and personal messaging.^21^

Furthermore, previously reported studies have elaborated that females have a tendency towards using more internet and have an inclination for an indirect communication in order to remain in touch with their friends, family, and colleagues.^22^,^23^

This study is important in that it provides several implications related to blended synchronous learning. Moreover, the importance of diversity of learning opportunities and improvement to academic grades has been shown. Easy and speedy transference of links to study materials via WhatsApp messenger not only increase student engagement of discussing the lecture content but also enhance students’ learning abilities and make them confident to return to the classroom with additional knowledge.

The findings of this exploratory study suggest the positive effect on the final grades and overall class attendance of the smaller groups. However, several confirmatory studies in different students at different campuses across the country should be carried out not only with a larger sample size but also in the context of more than one learning course to get a clearer picture. This is a pilot study that provides instrumental insight into teaching styles and methodologies. In the current demographic settings it was not possible for the authors to study a gender-neutral class. It is therefore suggested that further studies must be carried out to observe the outcomes of gender-neutral class. Additionally, other studies must focus to infer the differences if a single instructor teaches with different methodologies and approaches.

## CONCLUSION

The present exploratory study clearly validates the usefulness of WhatsApp messenger as an educational tool or learning platform for enhancing students’ knowledge and overall achievements in examination grades. Mobile learning through WhatsApp helps students to discuss more challenging topics or courses in the virtual environment. Through collaborative and networked processes, students who tend to be reserve-natured in the classroom setting may improve their knowledge compared to LMS, which is an administrative tool. In addition, sending text via WhatsApp messenger is an easy and cost-effective method to improve in-class attendance of the students. The outcomes of this study are important for educational practitioners in improving the connections among the students themselves as well as the relation between students and their tutors.
